# Psychosocial Needs Assessment among Earthquake Survivors in Lorestan Province with an Emphasis on the Vulnerable Groups

**DOI:** 10.5539/gjhs.v5n4p79

**Published:** 2013-04-07

**Authors:** A. Forouzan, M. Baradaran Eftekhari, K. Falahat, M. Dejman, N. Heidari, E. Habibi

**Affiliations:** 1 Social Determinants of Health Research Center, Welfare and Rehabilitation University of Medical Sciences, Tehran, Iran; 2 Undersecretary for Research and Technology, Ministry of Health and Medical Education, Tehran, Iran

**Keywords:** earthquake, vulnerable groups, psychosocial needs, Iran

## Abstract

**Introduction:**

Iran is one of the ten most earthquake prone countries in the world. Earthquakes not only cause new psychological needs among the population but particularly so when one considers vulnerable groups. This in - depth study was conducted with the aim of assessing psychosocial needs six months after an earthquake happened in the west of the county in Lorestan province.

**Methods:**

This is a qualitative study using focus group discussion that focuses mainly on the vulnerable groups (women, children, elderly and disabled people) after an earthquake in Boz-azna; a village in Lorestan province in western part of Iran.

**Findings:**

Results of the psychosocial assessment indicated feelings of anxiety and worries in four vulnerable groups. Horror, hyper-excitement, avoidance and disturbing thoughts were observed in all groups with the exception of the elderly. Educational failures, loneliness and isolation were highlighted in children. All groups encountered socio-economic needs that included loss of assets and sense of insecurity and also reproductive problems were reported in women's group.

**Discussion and Conclusion:**

Modification of a protocol on psychosocial support considering the context of the rural and urban areas with emphasis on the specific needs of the vulnerable groups is an appropriate strategy in crisis management. It seems that appropriate public awareness regarding assistance programs can be effective in reducing stress and needs of disaster survivors.

## 1. Introduction

For centuries, natural disasters and the ways of controlling them have been consistently considered by human beings. In the past decades, approximately 86% of the total deaths caused by disasters were attributed to natural disasters. Seventy five percent of deaths were Asians. Natural disasters cause an average annual loss of 87 billion dollars (Kahn, 2005)

Unfortunately, Iran is a country prone to natural disasters and is known as a one of the most vulnerable countries in the world. Approximately 21 types of 40 known natural disaster types in the world occur in Iran. Generally, Iran is considered one among the 10 most disaster-prone countries in the world (Khankeh et al., 2006). Occurrences of natural disasters such as earthquakes create special circumstances in the community and also create new needs. It can cause major changes in common and ordinary patterns of life and create specific mental and psychological conditions to encounter (Emami et al., 2005). A study conducted by Chon Ying-jeng and associates found that (2003) 2-15 months after an earthquake that happened in Taiwan, earthquake victims have committed suicide 46.1% more than the control group suggesting that the impact of that earthquake on the occurrence of suicide is statistically significant.

Results of the study conducted by Kato and colleagues (1995) for the purpose of assessing the mental effects of the earthquake in Hanshin Awaji in Japan showed that survivors experienced sleep disorders, depression and increased irritability. Anxiety disorders were the most common complication reported immediately following the earthquake which gradually declined a month after (Park, 1995)

Recommendations made by the World Health Organization (WHO) on the area of mental health has stated that psychosocial support for earthquake victims must be community-based and appropriate to the culture of the region and should also consider the needs of specific groups such as women, children, elderly, disabled etc (Van Griensven et al., 2006). Children and adults probably manifest similar problems after the incident; however, the method of expressing these problems could be completely different. Children's reactions to disasters can include feelings of anxiety, fear and concern of the occurrence of another earthquake and behavioral changes (Gurwitch, Silovsky, Schultz, Kees, & Burlingame, 2002).

Limited studies have been implemented concerning the disorders following a disaster in Iran and results of these studies have just provided a view of common injuries and needs of survivors. A study conducted in relation to mental disorders of survivors of an earthquake in the north of Iran –Rudbar– in 1990 showed that 68% of the subjects studied were affected with major depression of which 38% suffered severe types of depression (Berberian, Qorashi, Jackson, Priestley, & Wallace, 1992). Another study conducted among the children of that area 3 years after the earthquake found 60% demonstrated behavioral disorders which was 2.5 times more than the control group. Furthermore, 51.7% of these children (ages between 9-16 years) have suffered post-traumatic stress disorder (PTSD) (Diaz, Murthy, & Lakshminarayana, 2006).

Occurrences of mental illness years after natural diaster in children have had the same prevalence in adults. In a study conducted by Yasami and associates in the year 1997-1988, the mental pathology in the southern region of Khorasan and Ardebil were compared with the control group. In general, psychopathology among adults was 3 times higher and among the children 2 times higher than control group. About forty seven of children and 46.5% of adults suffered moderate to severe PTSD a year after the earthquake tragedy. Also, in the study conducted in Bam, 82% of adults and 84% of children (based on AHQ, Rutter scale) suffered from psychological problems. Based on Watson's scale, percentage of adults and children who suffered from PTSD were 65% and 78% (Ahmadi et al., 2011; Diaz, Murthy, & Lakshminarayana, 2006).

In this study, the authors have decided to conduct an in-depth qualitative study using a more detailed method in order to assess the psychosocial needs of specific groups (children, women, elderly and the disabled) based on their words after the earthquake in Lorestan in 2006. In this earthquake, three persons were killed in this village.

## 2. Materials and Methods

This qualitative study was conducted using focus group discussion. The reason for using this method is its speed in operation and in finding more information with the presence of more individuals with less time. Location of the study was in Boz-azna village, situated 25 kilometers from Boroujerd which during the earthquake that occurred on 11^th^ of March 2006, suffered 80% of the damages. Selection of individuals participating in group discussions was based on following criteria as; member of vulnerable groups and opinions of the healthcare providers of Boz-azna health center considering appropriate access to survivors respectively.

Target groups consisted of four vulnerable groups considered: (a) Women between the ages of 15 and 49, (b) children of both sex ages 11 to 14, (c) the elderly of both sex who were older than 60 years of age, and lastly, (d) the disabled of both sex. Participants of each focus group discussion were between 6 persons (in elderly) and 12 persons (in women). By using guide questionnaires, each focus group discussion was at least 90 minutes duration and the number of all focus group discussion was based on achieving data saturation. Each focus group discussion, with the permission of the participants, was recorded with the use of a tape recorder and transcribed at the end of each session. Data analysis was manually done using the inductive deductive content analysis method based on the study objectives.

For objectivity (conformability) of data, the following methods were used by the members of the team; researchers’ credibility, prolonged engagement, persistent observation, good communications with the overview of the supervisors (external check), use of additional comments from colleagues (peer debriefing) and reviewing of the hand-written notes. All ethical issues such as acquisition of consent from all participants of the focus group discussions, maintaining participant's anonymity, confidentiality and the right to withdraw anytime from the study have been taken into consideration.

## 3. Results

Results on the psychosocial needs of vulnerable groups after the Lorestan earthquake have been segregated and presented into the following manner: women, children, elderly and disabled people.

### 3.1 Psychological Needs

#### 3.1.1 Women

- Anxiety and worry *“I feel anxious and think at any time an earthquake will occur again and the scene of the earthquake would come rushing back into my imagination”*
- Depression *“I felt so alone and I’m depressed”*
- Fear “My sister hates to talk about the incident since she has felt more scared”
- Aggression *“People easily get angry and fight over something worthless”*
- Psychological pressure *“all mental pressures, work and take care of the children have been burdened by women, taking care of the children when you are homeless and living in tents”*

#### 3.1.2 Children

- Fear: *“We never thought that an earthquake would be so devastating, and at present we still feel scared. We were very scared specially when we saw those fatalities… we felt very sick*”
- Hyper arousal:*” My son would scream even to the slightest sound”*
- Educational failure: *“After the earthquake, the children were no longer motivated to study even those who were top in their class”*
- Depression: *“The children said that they lost their hope, depression is overwhelming, one child was crying due to the death of her grandmother*”
- Loneliness and isolation: *“Students feel lonely and isolated”*
- Disturbing thoughts: *“My thoughts are all about earthquakes, I see the incident in my mind”*
- Avoidance: *“no one wants to talk about earthquake or even repeat the word of earthquake”*

#### 3.1.3 Elderly

- Anxiety: *“I always think that the incident will happen anytime*”
- Fear: *“For 3 to 4 months I never went near my destroyed house, I was so terrified. we afraid of darkness”*

#### 3.1.4 Disabled

- Fear: *“We were frightened of our own house, everything was destroyed, and she always screened where her kids were, where her kids have gone? Under the collapse”*
- Anxiety: *“My child who had spinal cord damages bothered so much, it was terrible, he has become hyperactive and we are worried all the time”*
- Hyper arousal: *“We are even afraid of the loud sound of a car”*
- Disturbing thoughts: *“we brought him several times to the hospital overnight, thoughts of the death comes to his mind and it is so terrible”*
- Psychological pressures: *“she is so confused, she seems to be out of her mind and suddenly goes out of her home”*

### 3.2 Socio-economic Needs

#### 3.2.1 Women

- Loss of money and properties: *“it was a horrible experience, we suddenly lost our house”*
- Loss of comfort and convenience: *“We lived in a tent for many days and it was hard”*
- Need to security: *“During the night when we had to go out, we had to go in a group of 2-5 persons because we afraid of wolves and foxes that had eaten the chickens*.
- Reproductive problems:
  a- Insufficient contraceptive facilities *“for three days after earthquake, we didn’t have any pill for contraception”*.
  b- Interfere with spouse *“living in a tent and daily stress after earthquake, disrupted the personal and marital relationship”*

#### 3.2.2 Children

- Sense of insecurity- *“Our houses were destroyed and our toys were missing until a few days later, authorities gave new toys*
- Need to accessibility to educational services – *“After earthquake, we couldn’t find any book, we didn’t have school uniforms”*

#### 3.2.3 Elderly

- Loss of money and properties – “ *our village has been destroyed, our houses are not in good condition, our buildings have been destroyed, our animals are under the collapse, our household appliances have been gone, we have no more home furnishings*”
- Problems regarding comfort and convenience- “ *we were living in the tents for many days, we do not have anywhere to go*”, *“our houses are still under construction”, we had to live in tents until summer”*

#### 3.2.4 Disabled

- Economic problems: *“we suddenly lost all of things”*
- Need to access to welfare facilities*:” My husband is still sick and after earthquake I expected to have more help”. “we had to live in tents until summer and it was hard for disabled people”*
- Need to access to services for the disabled: *“ immediately after earthquake there wasn’t sufficient services for the disabled for example there was no special place prepared for them such as tents with toilet and bath facilities but after short time, they provided some of them”, “a personnel from the social welfare used to visit us once a month*

## 4. Discussion and Conclusions

In times of crisis such as earthquake, secondary effects create special needs for vulnerable groups such as women, children, elderly and disabled people. If these needs are not planned and managed properly, a broader and deeper tragedy may occur and the social aftershocks and deep psycho-social trauma would present their effects for years to come. Women who lost their husbands and assumed responsibilities towards for their siblings that survived on one hand and the violence, addiction and irresponsibility's of their husbands on the other hand, have been exposed to severe psychosocial traumas (Dasgupta, Siriner, & De, 2010). It seems that after the earthquake in Lorestan, women suffered more due to their husband's negligence; these women needed special support from stakeholders. Security needs, physical abuse and sexual harassment are the other threats which women face, especially in times of crisis (Dasgupta et al., 2010) which has not been reported in this earthquake. With regards to children, one of the fundamental problems that this group would encounter in such a situation is educational failure or dropping out from school (Hebblethwaite, 2012). The results of this study clearly indicate the presence of such symptoms in children of this area. Children have experienced fear and anxiety due to the loss of their family, fear of being alone and the fear of another earthquake. Problems associated with welfare facilities, lack of appropriate places to be used as classrooms (classrooms were used to keep animals), and, unavailability of books and other school materials have been considered to play critical roles in educational failure for students. Considering that the children did not understand the importance of keeping livestock for family livelihood, they were worried. Providing mental and emotional supports for families is one of the most important expectations of the children in this area. It is evident that providing appropriate education to children and their family before an earthquake will make this vulnerable group more prepared in dealing with a disaster.

Another vulnerable group is the elderly, who live alone and are unable to receive assistance from other sources and therefore will be more susceptible to psychosocial problems (WHO, 2005). An overview of the needs expressed by this group showed that socio-economic problems and loss of money and properties are the most difficult problems experienced by the elderly. In truth, the mere loss of things which they have gained throughout their lives would cause extreme trauma to this group and therefore, special attention is required to meet their demands.

The last groups of individuals which are considered vulnerable are the disabled which due to their multiple disabilities should be given more special welfare assistance (Chou et al., 2004). After earthquake, the most important action is rescuing survivors and providing the public health priorities, on the other hand in this area, there were only three disabled persons that taking support from their family could be useful to manage them Obviously, existing problems in addition to physical disability would result to more fragility during crisis and their need for more assistance whether financial or spiritual would increase. It seems that dispatching trained personnel and providing special welfare services with consideration of target groups needs, play an effective role in psychological rehabilitation.

Based on the policy, temporary accommodation in the Lorestan earthquake was removed. It was due to simple reconstruction in rural area and good climatic conditions. Although this decision had advantages and disadvantages, but enough information could support victims and reduce stress, especially in vulnerable groups (Omidvar, Ghasemi, & Zafari, 2008).

It seems that designing and implementing the new protocol regarding to psychosocial needs in vulnerable groups after disaster can be very useful in preventing the psychological problems.

Finally, it is important to note that participation of all vulnerable groups in social activities is extremely useful in regaining their lost identity and morale (WHO, 2005).
